# Experimental Analysis of Perimeter Shear Strength of Composite Sandwich Structures

**DOI:** 10.3390/ma14010012

**Published:** 2020-12-22

**Authors:** Łukasz Święch, Radosław Kołodziejczyk, Natalia Stącel

**Affiliations:** Department of Aerospace Engineering, Faculty of Mechanical Engineering and Aeronautics, Rzeszow University of Technology, al. Powstańców Warszawy 12, 35-959 Rzeszów, Poland; rkolodziejczyk@prz.edu.pl (R.K.); 154407@stud.prz.edu.pl (N.S.)

**Keywords:** composite structures, sandwich structures, static shear test, concentrated force on composite, experimental analysis, honeycomb, destruction process

## Abstract

The work concerns the experimental analysis of the process of destruction of sandwich structures as a result of circumferential shearing. The aim of the research was to determine the differences that occur in the destruction mechanism of such structures depending on the thickness and material of the core used. Specimens with a Rohacell foam core and a honeycomb core were made for the purposes of the research. The specimen destruction process was carried out in a static loading test with the use of a system introducing circumferential shear stress. The analysis of the tests results was made based on the load-displacement curves, the maximum load, and the energy absorbed by individual specimens. The tests indicated significant differences in the destruction mechanism of specimens with varied core material. The specimen with the honeycomb core was characterized by greater stiffness, which caused the damage to occur locally in the area subjected to the pressure of the punch. In specimens with the foam core, due to the lower stiffness of that core, the skins of the structure were bent, which additionally transfers compressive and tensile loads. This led to a higher maximum force that the specimens obtained at the time of destruction and greater energy absorption.

## 1. Introduction

The progressive development of technology in the field of construction of load-bearing structures is currently focused mainly on improving the properties of the used materials and the use of modern structural solutions. This has a number of benefits, such as increasing stiffness, strength, improving fatigue characteristics, and finally, reducing the mass of such structures [1]. These features are important in many fields of technology, such as automotive, aviation, and space technology [2,3]. It is particularly desirable to achieve all of the listed benefits simultaneously. Such possibilities are created using sandwich structures. Despite the fact that they have been used for a long time, the mentioned development of materials resulted in the fact that the achieved benefits of their use are even more emphasized. So far, extensive studies of sandwich structures have been carried out, considering a multitude of ways of loading such structures as well as methods of their analysis and modeling [4,5,6]. Sandwich structures demonstrate high bending [7,8], compressive [9,10], and shock load strength [11,12,13,14]. However, concentrated loads and the way they are introduced into the composite structure have crucial importance for its operational durability [15,16,17,18,19]. Among the whole range of possible analyses to which such structures can be subjected, its damage under the influence of perpendicularly applied concentrated load is noteworthy from the authors’ point of view. The application of certain special design solutions may result in the occurrence of such loads. This is particularly evident in the area of fasteners, such as, for example, wing-fuselage fittings in aircraft construction. The method of introducing concentrated loads is still a current problem, and the analysis of such a case may allow for the formulation of guidelines for the selection of materials in this type of solutions. Another source of concentrated loads may be incorrect exploitation. They are also often the result of random events. Examples include a collision with a bird/drone of a sandwich structure of a wing or an aircraft fuselage, a collision with an obstacle of a car with a sandwich body, or a collision with rocks of the boat hull, among others. Such incidents often result in severe structural damage and are sometimes associated with costly repairs. The method of destroying such structures becomes important here. Knowledge of this mechanism allows for the targeted selection of materials used in its construction as well as the proper design of its geometry. For example, the destruction of a racecar’s body structure should be different from the destruction of the leading edge of an airplane wing as a result of a collision with an object. The first case is a structure which should deform over the entire surface and absorb as much of the impact energy as possible. In the second situation, the failure should be local and should not affect the geometry of the structure, which is not under the impact of the destructive load. The main goal of the analyses is to understand the abovementioned destruction mechanism of sandwich structures, as well as the determination of possibilities of their absorption of energy.

The paper analyzes the method of destruction of the sandwich structure exposed to circumferential shear depending on the core material used in this structure. The results of similar studies can be found in the literature [20]. However, they have not analyzed the mechanism of destruction of the considered structures. There are numerous publications describing the mechanism of destruction of sandwich structures, but they differ from the presented case in the way that the loading is introduced to such a structure [21,22,23,24].

Sandwich composites are classified as structural composites [25,26,27]. Their structure is characterized by the presence of two thin facesheets with a light core between them. There are a number of different types of core solutions in sandwich materials, primarily foams, balsa, or honeycomb structures, as well as other solutions [28,29,30,31,32,33]. The core is a filling, which increases the moment of inertia of the cross-section of such a structure by moving the two outer skins away from each other [34]. This leads to an increase in the flexural stiffness in the plane of such a load-bearing structure. Furthermore, the core is intended to transfer shear loads and provide compressive strength in the direction perpendicular to the surface of the sandwich structure [35,36].

The structure analyzed in the paper has skins made of carbon composites. The properties of such layers depend on the properties of their component elements and the quantitative ratios of these elements. They are also highly sensitive to the manufacturing process and the arrangement of subsequent layers of composite reinforcement. This requires more research than in the case of traditional materials to obtain their full characterization. One of such tests is the circumferential shearing of the sandwich structure presented in the paper. The destruction mechanism of the sandwich structure in such a test is strongly dependent on the core material used and its thickness [37].

## 2. Materials and Methods

**Figure 1:**
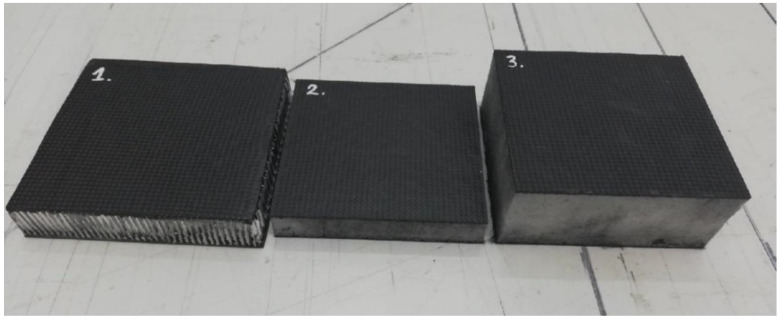
Specimens prepared for tests.

Three specimens of the sandwich structure were tested in the study. They are presented in Figure 1. All specimens had carbon composite skins. The configuration of the structure layers was the same for each of the specimens and the only difference was the core material used. The facesheets had a symmetrical arrangement of layers in the configuration (45/90/0/90/45). The layers oriented at an angle of 45 degrees were made of a plain weave fabric with a grammage of 200 g/m^2^. The other layers were made of unidirectional fabric with a grammage of 250 g/m^2^. The dimensions of the specimens in the plane were about 100 mm × 100 mm.

Specimen number 1 (numbering according to Figure 1) had a core made of a 25 mm thick aluminum honeycomb structure. The other 2 specimens were filled with Rohacell foam with thicknesses of 20 mm and 50 mm for specimen 2 and specimen 3, respectively. The composite skins were made using the vacuum bag method and then heated at the temperature of 80 °C to improve the strength properties of the composite and reduce the time of full curing of composites. The MGS L285 (Havel Composites CZ Ltd., Svésedlice, Czech Republic) epoxy resin with the H287 (Havel Composites CZ Ltd., Svésedlice, Czech Republic) hardener was used as the matrix. The mechanical properties of this resin are presented in Table 1.

The facesheets of the sandwich structure were joined with the core material using MGS L285 resin with H285 hardener and aerosil. Typical values of mechanical properties for the used core materials are presented in Table 2.

**Figure 2:**
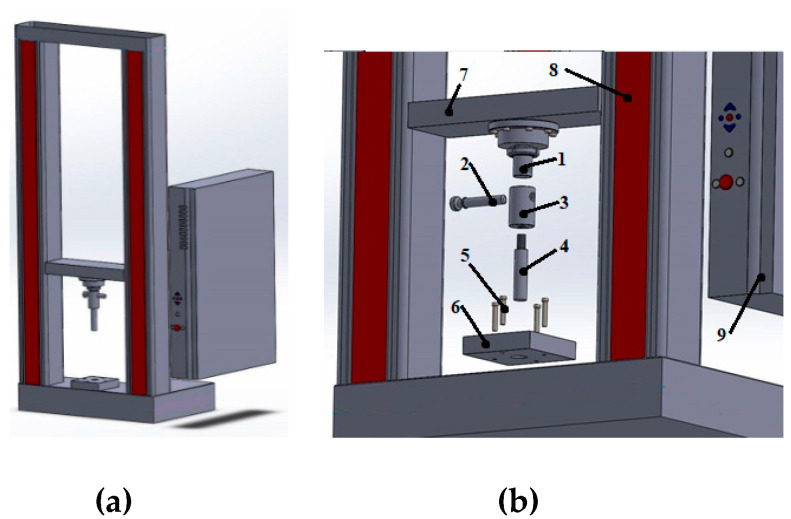
Test stand diagram. (**a**) General stand diagram; (**b**) Elements of the loading system, 1—force measuring head, 2—fixing pin, 3—bushing, 4—punch, 5—bottom plate mounting bolts, 6—bottom plate with 32 mm hole, 7—machine upper crosshead, 8—machine frame, 9—control electronics.

**Figure 3:**
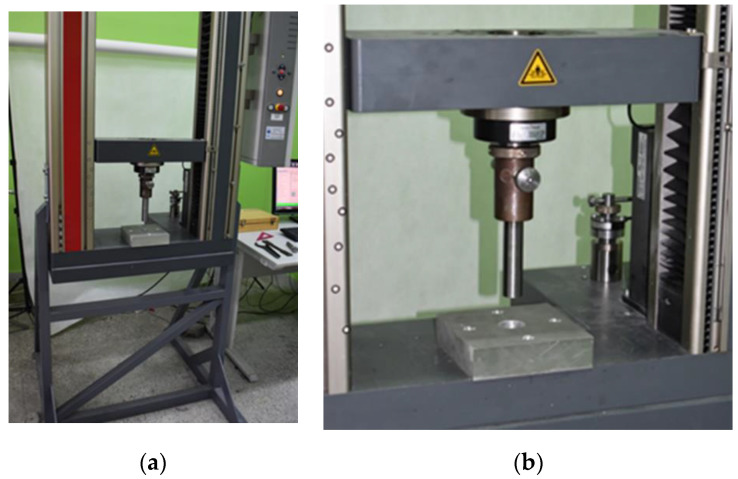
Technical implementation of the test stand. (**a**) General view of the stand; (**b**) Loading system for circumferential shearing of specimens.

The test of circumferential shear of the specimens was carried out with the use of the Zwick Roell Z050 (ZwickRoell GmbH &Co.KG, Ulm, Germany) testing machine. On the movable upper crosshead of the machine, there was a 25 mm diameter punch, which introduced shear loads to the test specimen during the movement of the crosshead. The specimens were placed on a bottom plate with a 32 mm diameter hole. After puncture of the sandwich structure, the punch could move freely through this hole. The tests were carried out with the displacement control with a constant speed of crosshead displacement equal to 3 mm/min. The diagram of the test stand is presented in Figure 2. The technical implementation of the stand is presented in Figure 3.

## 3. Results

As a result of the conducted tests, all the specimens were destroyed, and the force-displacement characteristics were recorded. Moreover, photographic documentation was made during the test showing the subsequent stages of specimen destruction. The results of the tests are presented below in the form of graphs. The number of markers in the graphs correspond to the numbering in the pictures showing successive stages of the test.

### 3.1. Results for Specimen Number 1

**Figure 4:**
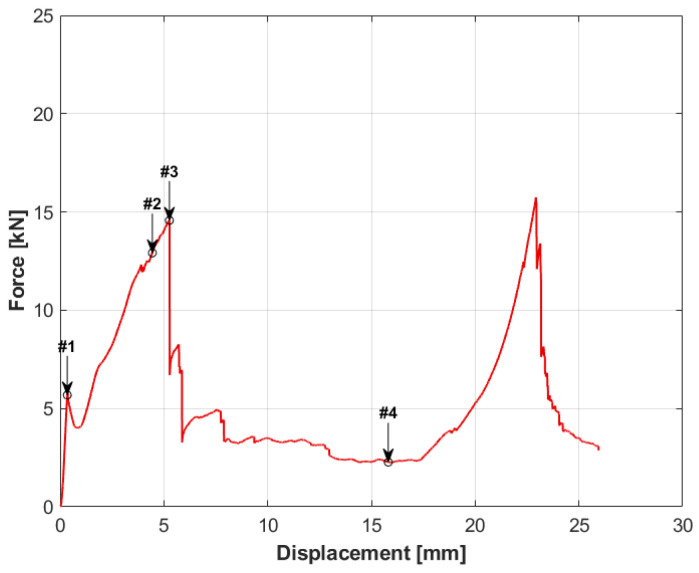
Force-displacement graph for specimen number 1.

**Figure 5:**
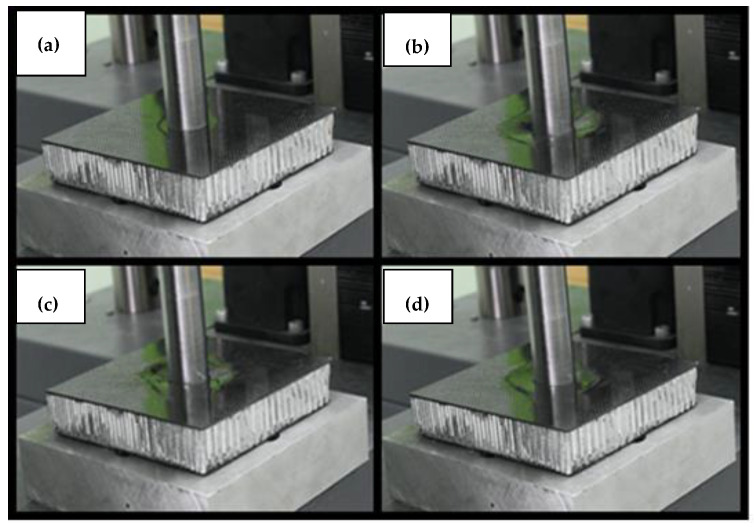
Pictures from the destruction process of specimen number 1. The numbering (**a**–**d**) in the subfigures refers to the markers in the graph in Figure 4 and present the successive stages of loading.

The test results for the sandwich structure with the aluminum honeycomb core are presented in the graph in Figure 4, and the movie captured during the test is available as the supplementary file (Video S1). In the initial loading phase, a rapid increase in force can be observed along with the punch displacement, up to the value of 5.8 kN. After reaching this value, a sudden drop in force occurred, which was most likely caused by local detachment of the skin from the core. This point is marked on the graph as #1. It is presented with analogous numbering in Figure 5. Further loading of the specimen resulted in an increase in force, however, with a smaller gradient than initially, until the maximum value of 14,576 N was reached. This point was marked as #3. The applied load resulted in a puncture of the upper skin of the specimen, after which the force dropped sharply. In the further phase called the “plateau,” the moving punch acted directly on the core, causing its compression and destruction (point #4). Shear force at this stage slowly decreased from 3.5 kN to 2.3 kN. This phase was followed by another sudden increase in force due to the fact that the lower skin began to carry the shear load. The core was damaged, and it was no longer able to absorb energy. It is worth noting that the increase in force started after the punch displacement of 17.5 mm, while the core material was 20 mm thick. This is because the material from the upper skin and compressed core had accumulated in front of the moving punch. It is also visible in the changing force gradient in the last phase, which was the result of further compression of the material accumulated in front of the punch. After reaching the second peak of the force of 15,744 N, the lower skin was punctured.

**Figure 6:**
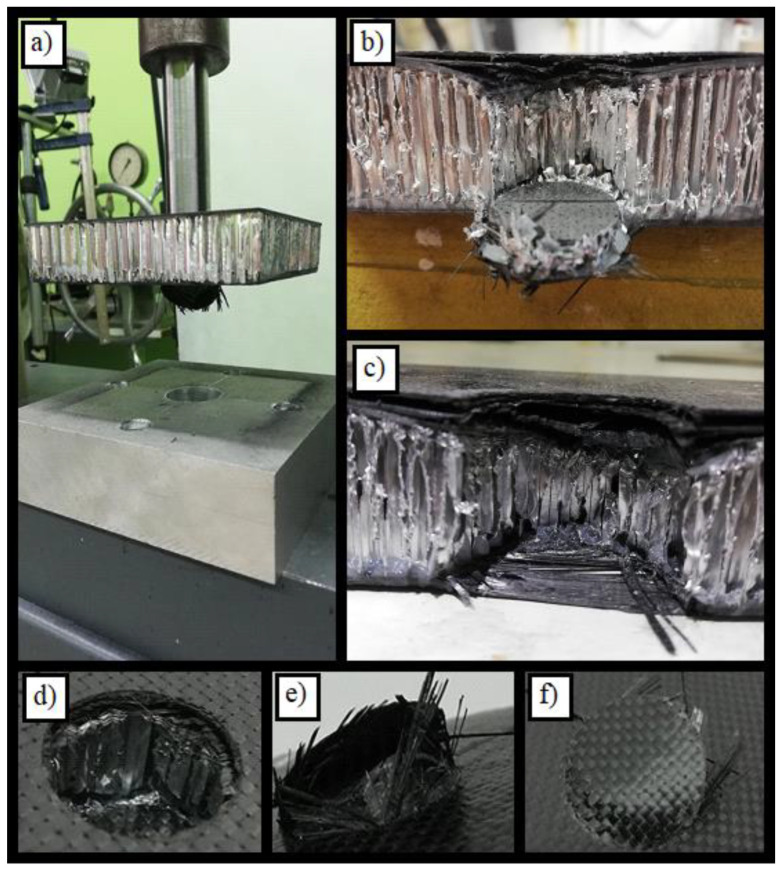
Pictures showing the destruction of specimen no. 1 after the test. A detailed description of subfigures (**a**–**f**) in the text.

The effects of specimen destruction can be observed in Figure 6. The picture marked with letter d in Figure 6 shows the hole which formed in the upper skin. As can be seen, this hole had smooth edges and a circular depression was formed around it. Figure 6b,c show the cross-section of the resulting hole and the effect of the destruction of individual layers of the sandwich structure. The delamination of the upper skin is also visible here. The material of individual layers accumulated in the hole, shown in Figure 6b, is noteworthy. The damaged lower CFRP facesheet is shown in Figure 6e,f. As in the upper skin, the regular circular edge of the hole is visible here, except the fact that this edge has been frayed. Some of the composite fibers were pulled out of the matrix. It is not without significance that the damage in this specimen was concentrated around the hole. Visual examination of the specimen shows that the areas distant from the stamp did not deform or that this deformation was imperceptible. Possible future tests should include the measurement of deformation of the areas outside the punch.

### 3.2. Results for Specimen Number 2

**Figure 7:**
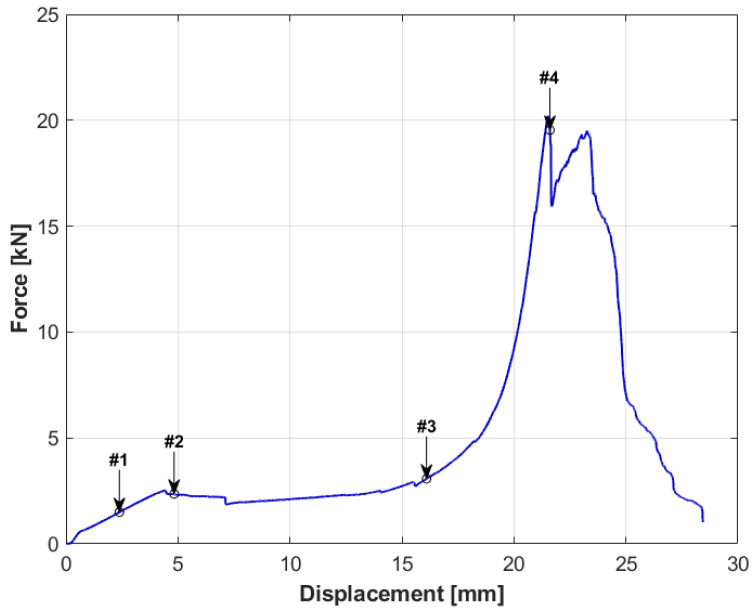
Force-displacement graph for specimen number 2.

**Figure 8:**
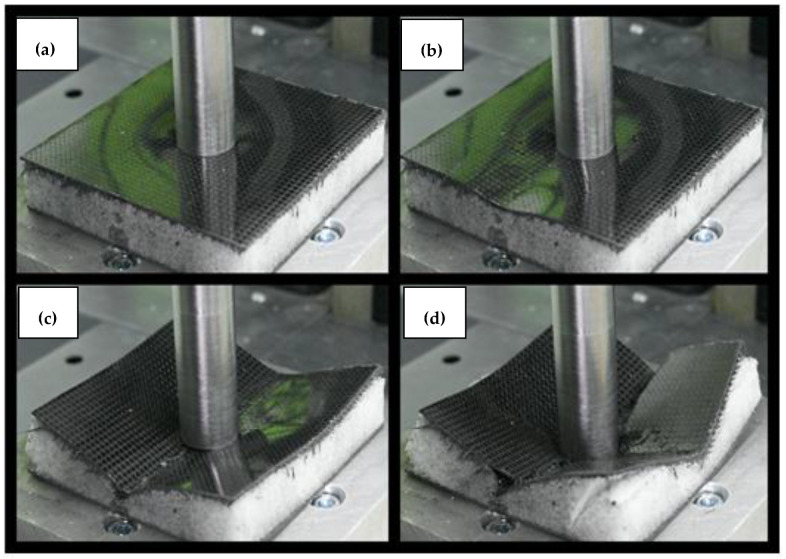
Pictures showing the state of destruction of specimen no. 2 during the test. The numbering (**a**–**d**) in the subfigures refers to the markers in the graph in Figure 7 and present the successive stages of loading.

The specimens with the Rohacell core had a different damage characteristic compared to the first specimen. The characteristics for the specimen with a 20 mm core are shown in Figure 7, and the movie captured during the test is available as the supplementary file (Video S2). The applied load caused the destruction of the specimen over its entire area, which resulted from the compression of the core and the significant difference in the stiffness of the materials from which the specimen was made. It is important that the plateau phase occurred even before the puncture of the specimen. The initial compression was completed with achieving the peak stresses of the core, followed by a slight decrease in force, as marked in the graph from Figure 7 as #2. This point was followed by a uniform compression of the core (plateau) to the point marked as #3. Then, the skins of the sandwich structure were destroyed, which, at this point, were very close to each other. The first (upper) skin was punctured with a sheer force of 20,207 N, followed immediately by the destruction of the second skin with a force of 19,505 N, as shown in the graph as two consecutive peaks. The first peak is marked as #4. This moment is presented in Figure 8 using analogous numbering.

By analyzing the pictures taken during the test sequentially, it can be seen that the initial behavior of the specimen was partially similar to specimen number one. It resulted in a circular deformation around the punch (Figure 8 picture #1). This stage is shown in the graph in Figure 7. However, it was characterized by a much greater increase in displacement with force growth than in the case of specimen 1. The similarity of both tests ends here. Then, after reaching the peak stress, the entire specimen began to break along the axis of the lowest stiffness, as can be seen in picture #2. The orientation of the specimen breaking direction depended on the arrangement of the skin layers. The resulting crack in the skin changed the manner in which the specimen was loaded, because the punch did not fully contact the surface of the sandwich structure. In the final step, picture #4, a second fracture, formed on the upper skin, can be observed along the second principal direction of the stiffness of the composite.

**Figure 9:**
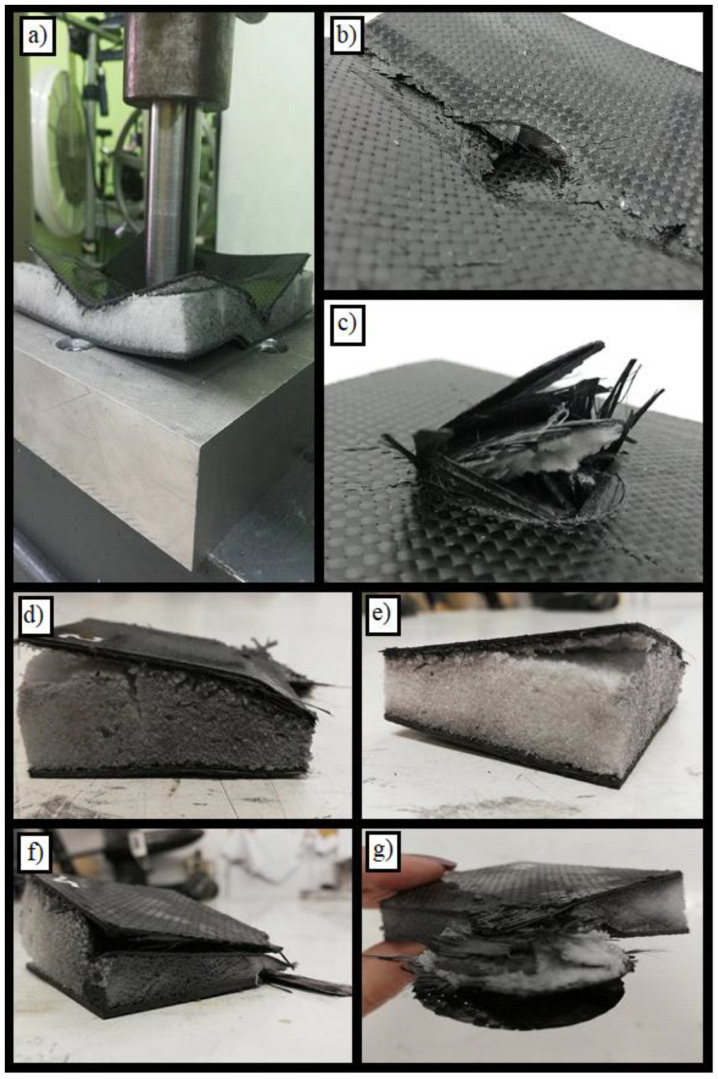
Pictures showing the destruction of specimen no. 2 after the test. A detailed description of subfigures (**a**–**g**) in the text.

The damaged specimen is presented in the series of pictures shown in Figure 9. A general view of the damaged specimen is presented in Figure 9a and the hole created by the punch can be seen in Figure 9b. It can be seen that the hole had an irregular shape that differed from that obtained in the first test. Moreover, the fracture along the specimen and the delamination area are clearly visible here. The bottom skin was damaged in a similar way to specimen number 1 (Figure 9c). Figure 9d–f show how the specimen deformed. There are visible areas where the core was torn as a result of deformation of the upper skin. Figure 9g shows a cross-section of the specimen with clearly visible material compressed by the punch.

### 3.3. Results for Specimen Number 3

**Figure 10:**
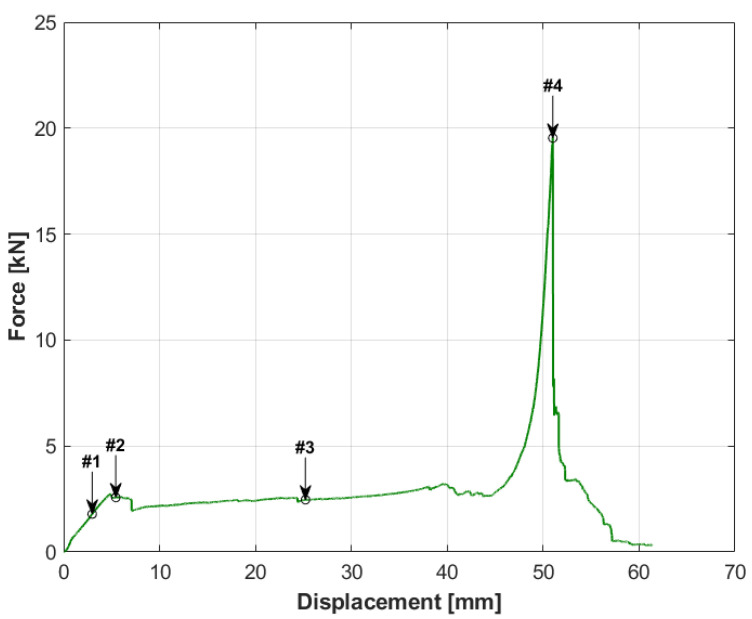
Force-displacement graph for specimen number 3.

**Figure 11:**
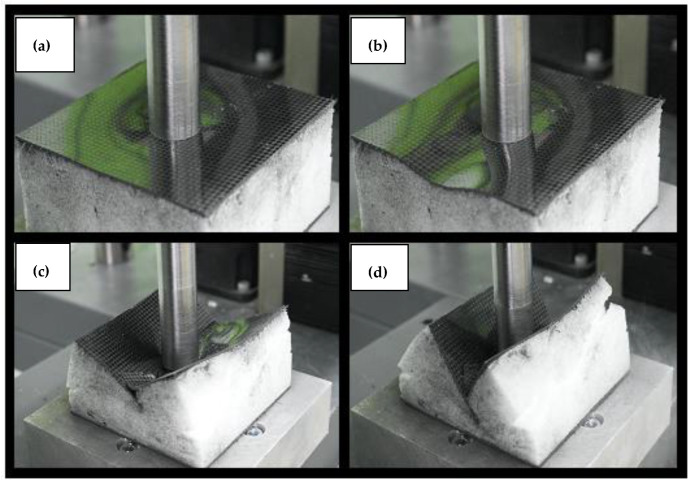
Pictures showing the state of destruction of specimen no. 3 during the test. The numbering (**a**–**d**) in the subfigures refers to the markers in the graph in Figure 10 and present the successive stages of loading.

**Figure 12:**
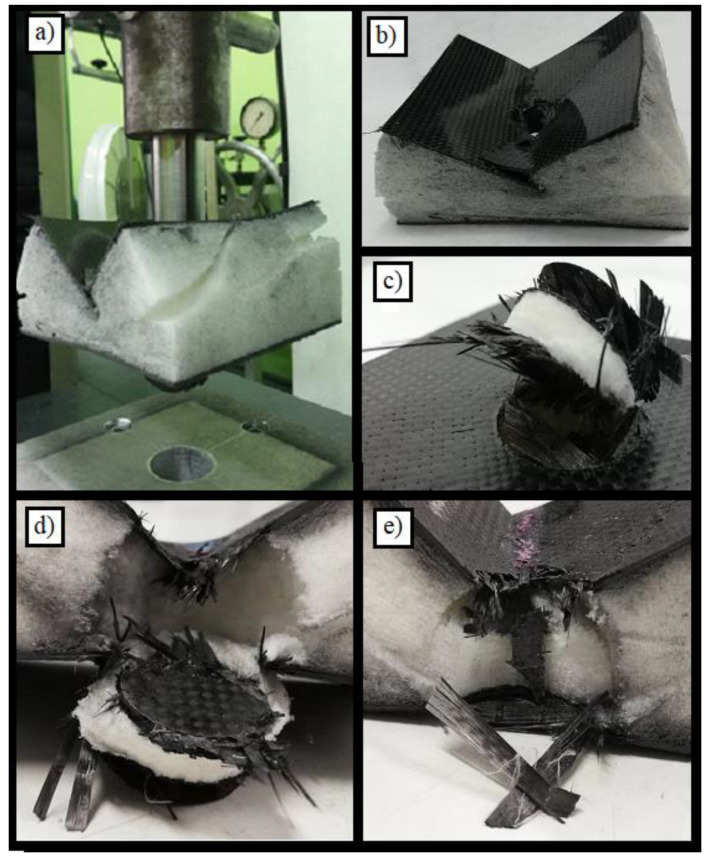
Pictures showing the destruction of specimen no. 3 after the test. A detailed description of subfigures (**a**–**e**) in the text.

The specimen filled with a 50 mm thick Rohacell core had comparable damage characteristics to specimen number 2. The main difference was a longer plateau phase due to the thicker core and the single peak force where the specimen was destroyed. This peak was achieved for the force of 19,572 N, which is comparable to that in test number 2. The test results are presented in form of a graph in Figure 10 and in the form of photographic documentation in Figure 11 and Figure 12. Additionally, the movie captured during the test is available as the supplementary file (Video S3). The general evaluation of the obtained results in the form of pictures indicates that the destruction mechanisms of specimen two and three were very similar. An important difference is that the upper skin of specimen number 3 was cracked only in one direction. This was probably due to the fact that, at this core thickness, the first crack in the direction of the lowest stiffness resulted in the destruction of the upper skin before the punch moved to the bottom skin. Consequently, the upper facesheet was no longer bearing load. This would explain the occurrence of a single peak in the graph in Figure 10.

### 3.4. Results Comparison

**Figure 13:**
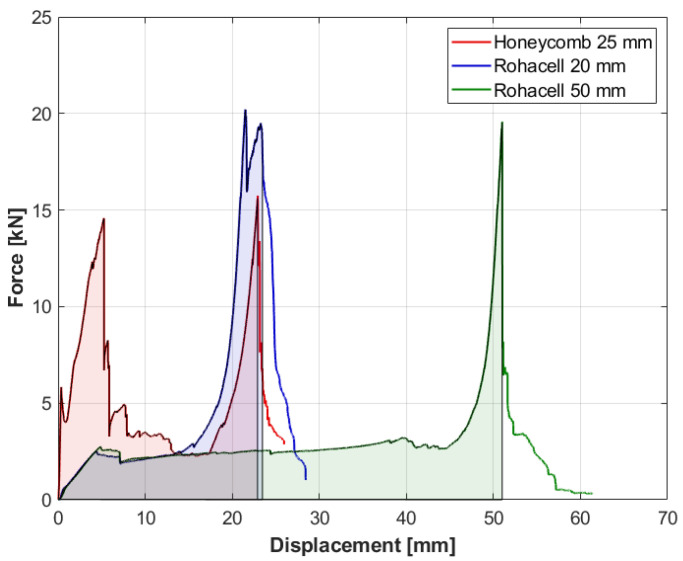
Force-displacement summary graph for all specimens. The shaded area represents the energy absorbed by the specimens up to the destruction point.

For comparison purposes, the results obtained during tests are presented collectively in the graph in Figure 13. The maximum values obtained for individual specimens are given in Table 3. It is worth noting that comparable maximum values were obtained for the specimens with the Rohacell core despite their different thicknesses.

The quantitative comparison of the obtained results was made on the basis of the energy absorbed by the specimens during the experiment. It was determined based on Equation (1):

(1) $$E_{A}=\int Fdl=\sum_{i=1}^{n}\frac{\left(F_{i}+F_{i+1}\right)}{2}\times \Delta l$$

The absorbed energy for each specimen is shown as the shaded area below the graph (Figure 13). Numerical integration was performed from the beginning of the test to the last force peak, showing the destruction of the specimen. The obtained results are presented in the table below (Table 4). Additionally, these results were referred to the core thickness (δ) of each specimen and its density (ρ).

The conducted analysis shows that specimen number 3 (Rohacell 50mm) had the greatest potential for energy absorption. However, since the specimens had different thicknesses, the comparison was made on the basis of the energy absorbed per unit thickness of the individual cores. This comparison shows that the specimen of the thinnest core (specimen number 2) had the highest ratio of energy absorbed to thickness. There was also a visible decreasing tendency in this parameter along with an increase in the thickness of individual cores. Comparing the specimens with the Rohacell foam core, it can be seen that a 2.5-fold increase in the core thickness caused a decrease in E_A_/δ parameter by 52%. However, it is difficult to say for sure whether the observed trend is right. A broader scope of research should be conducted to make the conclusions more probable. Quantity E_A_/ρδ is defined by the ratio of absorbed energy in relation to the mass of the sandwich structure per unit of area of this structure. A comparison of this quantity indicates a significant advantage of the foam cores over the honeycomb core in terms of mass reduction.

## 4. Discussion

On the basis of the presented tests and analyses of the obtained results, a comparison of the destruction process of the sandwich structures with three different cores was made. The most significant differences were seen between the first specimen containing an aluminum honeycomb core and the other two specimens having a foam core of different thickness. In the case of the first specimen, the lowest values of the maximum shear force were obtained, as shown in Figure 13 and in Table 3. The destruction of the sandwich structure of the first specimen was characterized by local damage around the punch which was loading the specimen. The area at the edge of the specimen, located within 50 mm from the punch axis, was not subjected to deformations. The destruction occurred gradually, successively destroying the upper skin, the core, and in the last phase, the lower skin. Therefore, the individual layers of the sandwich structure were mainly subjected to shear loads. The greater stiffness of the honeycomb core compared to the foam was responsible for the obtained result. The two remaining specimens were characterized by a crack and a bend of the upper skin along the axis of the lowest stiffness. This process, caused by significant compression of the foam, resulted in the fact that, in addition to shear loads, the skins also transferred compressive and tensile loads from bending, which ultimately increased the maximum force that was achieved in the test compared to the first test. Additionally, specimens with foam core deformed over their entire surface. This resulted in greater energy absorption than in the first specimen and caused the core material to break. Therefore, in future studies, it is advisable to compare the destruction of larger-sized specimens to see if the size effect may be significant for this process.

Comparing the specimens with the foam core of different thicknesses, it can be concluded that increase in the thickness caused elongation of the plateau area, but in this case, it had no significant effect on the maximum force obtained in the tests. However, it should be noted that such a conclusion was drawn on the basis of only two tests and may only apply to a certain range of specimen thicknesses. This indicates the necessity to conduct tests for a wider spectrum of different thicknesses of the core materials.

The performed tests differed from those found in the literature in terms of the method of introducing the load. Similar tests have been described by the authors of [20], but the specimens used differ from those used in this work in terms of dimensions and configuration of the composite layers of the sandwich structure. These tests also do not consider the use of a core material other than aluminum honeycomb. Hence, the quantitative comparison of the results is difficult. However, when analyzing the force-displacement diagram, a similarity in the formation of the load plateau, which is due to the compression of the core, can be noticed. An important difference in the results obtained in this study is that, in the case of the honeycomb core, there was an additional load peak showing the puncture of the top covering. In the case of work performed by the authors of [20], there was no such effect, and the nature of this chart is more similar to the examples with the foam core. The work performed by the authors of [22] compares the destruction of a sandwich structure with a different core due to three-point bending. Despite the different way of loading than in this study, some similarities can be noticed, including the formation of two load peaks in specimens with the honeycomb core. An analogous three-point bending test was performed by the authors of [13], but the effect of creating a double peak load was not observed. This effect was likely due to the different stiffness ratios of core and coverings in the compared cases, as well as different dimensions of specimens. It is also worth noting the difference in the load ratio in the plateau and in the peak. In the analyzed cases in this study, ratio was of the order of 17–20%, and the work performed by the authors of [20], the ratio was about 65–70%. These differences indicate the necessity of differentiating the material, number, and configuration of layers of coverings of the sandwich structure for different thicknesses and core materials in future research. They can also be caused by the scale effect which, again, indicates the need to vary the dimensions of the specimens in the planned future work. This approach is also confirmed by the destruction effect obtained in the tests on specimens with the foam core, which deformed over their entire surface.

## Figures and Tables

**Table 1:** Mechanical properties of MGS L285 resin with H287 hardener [38].

**Density**	(g/cm^3^)	1.18–1.20
**Flexural Strength**	(N/mm^2^)	110–120
**Young’s Modulus**	(kN/mm^2^)	3.0–3.3
**Tensile Strength**	(N/mm^2^)	70–80
**Compressive Strength**	(N/mm^2^)	120–140
**Impact Strength**	(kJ/m^2^)	45–55

**Table 2:** Mechanical properties of core materials [39,40].

Mechanical Properties	Unit	Honeycomb Aluminium	Rohacell 31 IG-F
Density	(kg/m^3^)	130	32 ± 7
Compressive strength	(MPa)	7	0.4
Compressive longitudinal elastic modulus	(MPa)	-	17
Compressive strength	(MPa)	3.38	-
Shear strength in the plane along the longitudinal direction	(MPa)	4	2.4
Shear modulus in the plane along the longitudinal direction	(MPa)	550	13
Shear strength in the plane along the transverse direction	(MPa)	2.5	2.4
Shear modulus in the plane along the transverse direction	(MPa)	350	13
Tensile strength	(MPa)	-	1
Tensile longitudinal elastic modulus	(MPa)	-	36
Operational temperature	(°C)	from −55 °C to 177 °C	Up to 130 °C

**Table 3:** Summary results of the conducted research.

Specimen	1st peak		2nd peak	
	Force (N)	Displacement (mm)	Force (N)	Displacement (mm)
Specimen 1 (honeycomb 25 mm)	14,576	5.27	15,744	22.93
Specimen 2 (Rohacell 20 mm)	20,201	21.53	19,505	23.27
Specimen 1 (Rohacell 50 mm)	19,572	51.01	-	-

**Table 4:** Quantitative comparison of results.

Specimen	E_A_ (J)	E_A_/δ (J/mm)	E_A_/ρδ (J m^2^/kg)
Specimen 1 (honeycomb 25 mm)	123.7	4.95	38.1
Specimen 2 (Rohacell 20 mm)	125.7	6.29	196.4
Specimen 1 (Rohacell 50 mm)	150.4	3.01	94.0

## Data Availability

The data presented in this study are available on request from the corresponding author.

## Supplementary Materials

The following are available online at https://www.mdpi.com/1996-1944/14/1/12/s1, Video S1: Destruction of specimen 1, Video S2: Destruction of specimen 2, Video S3: Destruction of specimen 3.
